# Development of a COSMO-SAC Parametrization with Advanced QM Method TZVPD-FINE

**DOI:** 10.1021/acs.iecr.5c01146

**Published:** 2025-07-14

**Authors:** Edgar T. de Souza, Murilo L. Alcantara, Paula B. Staudt, João A. P. Coutinho, Rafael de P. Soares

**Affiliations:** a Virtual Laboratory for Properties Prediction (LVPP), Chemical Engineering Department, 28124 Federal University of Rio Grande do Sul, Rua Ramiro Barcelos, 2777, Porto Alegre, RS CEP 90035-007, Brazil; b CICECO-Aveiro Institute of Materials, Chemistry Department, University of Aveiro, Aveiro 3810-193, Portugal

## Abstract

Accurate prediction of interactions between compounds is essential for designing advanced materials and industrial processes. COSMO-based models have become prominent tools for estimating phase equilibria in complex systems. In this work, a new COSMO-SAC parametrization is proposed using a more robust quantum mechanical approach: the Becke and Perdew functional with triple-zeta valence polarization with diffuse functions combined with a fine grid marching tetrahedron cavity (BP-TZVPD-FINE). This level of theory enables a refined description of molecular and ionic charge densities. Implemented in JCOSMO software, this parametrization supports input files from TURBOMOLE, improving accessibility for nonexpert users. The model’s performance was evaluated using 6977 experimental data points of infinite-dilution activity coefficients and vapor–liquid and liquid–liquid equilibrium data. Compared with previous parametrization, the new model demonstrated improved accuracy, particularly for systems involving amines, ethers, and dipolar aprotic solvents.

## Graphical abstract

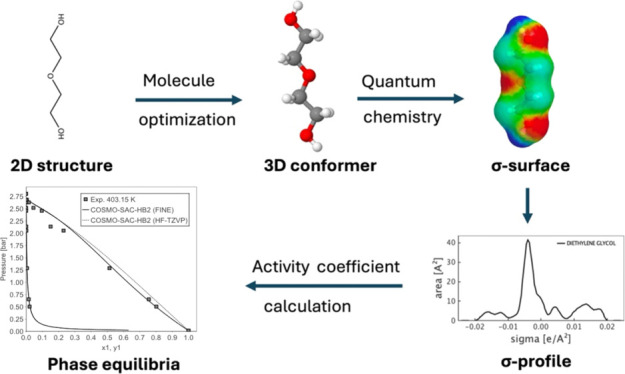

## 1 Introduction

As industries move toward more sustainable operations, the need for innovative and environmentally friendly processes has never been greater.^1^ Developing such processes demands precise and predictive tools that minimize the reliance on exhaustive experimental data, reducing costs and accelerating development. Predictive models are instrumental in this regard, not only enabling accurate predictions of complex systems but also providing insights into the underlying phenomena, aiding in both process design and a deeper fundamental understanding. Specifically, thermodynamic modeling is central to advancing research and development within the process of engineering and remains essential to designing new separation technologies that meet modern sustainability requirements.^2^

Phase equilibrium predictions are extremely important for efficient separation operations across various industrial applications, from pharmaceuticals to petrochemicals and beyond.^3^ Traditional activity coefficient models, including the nonrandom two-liquid (NRTL),^4^ Wilson,^5^ and universal quasichemical models (UNIQUAC),^6^ calculate the nonideality of liquid mixtures. However, these models often rely on extensive experimental data from binary systems, limiting their predictive capacity for novel or unconventional compounds. Predictive models like the universal quasichemical functional-group activity coefficient model (UNIFAC)^7^ attempt to address this issue but are restricted by the chemical groups and interactions for which parameters are available. While more robust molecular dynamics (MD) simulations^8^ can achieve highly accurate predictions, they often come with a prohibitive computational cost, which may not be feasible for all industrial applications. Activity models based on the conductor-like screening model (COSMO) have emerged as an alternative, offering a balance between predictive accuracy and computational efficiency. They enable the determination of activity coefficients without the direct need of experimental data.^9,10^

COSMO-based methods include the conductor-like screening model segment Activity coefficient (COSMO-SAC) model,^10^ which is a derivative of the original conductor-like screening model for real solvents (COSMO-RS) model.^9^ These models are useful for predicting the behavior of complicated systems, such as aqueous solutions and ionic liquids.^11–14^ The COSMO-SAC model assigns each molecule an apparent surface charge density distribution using the COSMO^15^ technique. To simplify mathematics, charge densities are typically represented by a single variable function called the σ-profile. The σ-profile shows a correlation between the fraction of the molecule surface and its charge density. To determine activity coefficients using COSMO-SAC, the σ-profile, area, and volume of each molecule must be known. This aspect makes the model predictive, as it relies only on a limited set of universal parameters.^16^

**Table 1:** Selected Substances for the Optimization of the Global Parameters according to Previous Works

chemical function	substances
hydrocarbons	saturated: 2,2,3-trimethylbutane; 2,2,4-trimethylpentane; 2,3,4-trimethylpentane; 2,4-dimethylpentane; 2-methylpentane; cyclohexane; methylcyclohexane; *n*-decane; *n*-heptane; *n*-hexadecane; *n*-hexane; *n*-nonane; *n*-octane; *n*-pentane
	unsaturated: 1-butene; 1-heptene; 1-hexene
	aromatics: benzene; toluene
organic halides	1,2-dichloroethane; chloroform; carbon tetrachloride
ketones	acetone; methyl-ethyl-ketone
esters	methyl acetate; ethyl acetate
ethers	diethyl ether; diisopropyl ether; dimethyl ether; tetrahydrofuran; methyl *n*-butyl ether
nitriles	*n*-butyronitrile
aldehyde	isobutyraldehyde; *n*-butyraldehyde
amines	aniline; triethylamine
alcohols	ethanol; *n*-butanol
others	water

**Figure 1:**
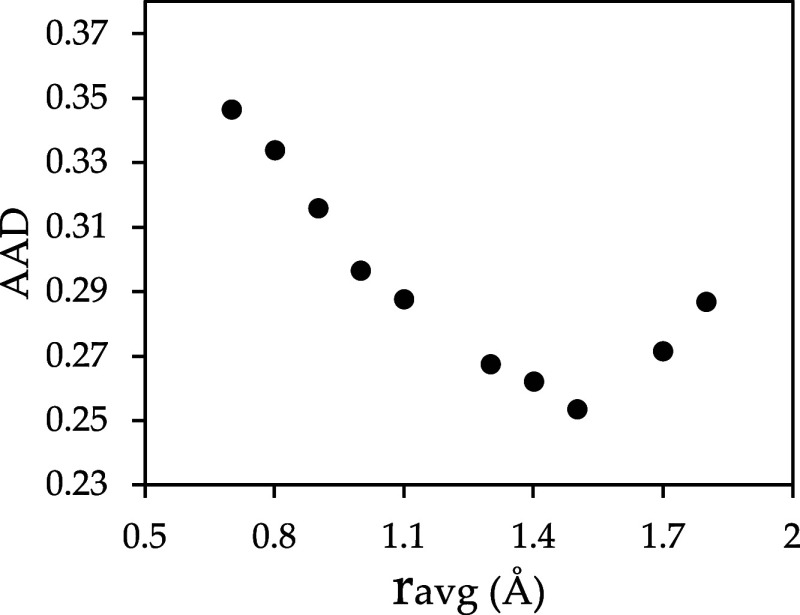
Plot of the average absolute deviation (AAD) as a function of the average radius using FINE and COSMO-SAC-HB2.

**Table 2:** Estimated COSMO-SAC Global Parameters^a^

	COSMO-SAC-HB2 (FINE)	COSMO-SAC-HB2 (BP-TZVP)	COSMO-SAC (FINE)	COSMO-SAC-HB2 (HF-TZVP)	COSMO-SAC (HF-TZVP)
*f*_pol_	0.9987	0.7560	0.8893	0.7817	0.8041
*r*_avg_ (Å)	1.5000	1.5000	1.6000	1.1000	1.4000
*r*_eff_ (Å)	1.1781	1.2806	1.2547	1.1567	1.1565
*C*_HB_	8263.93	8400.52	181,543.18	15,020.48	58,250.4900
*C*_HB2_	16,061.92	17,251.80		14,171.02	
*C*_HB3_	7496.26	11,123.03		9327.21	
*C*_H__B4_	16,989.74	17,575.78		14,171.02	
*C*_HB5_	25,332.78	23,721.46		14,171.02	
*C*_HB6_	12,882.18	12,733.48		6866.67	
*C*_HB7_	28,400.07	28,538.48		4642.64	
*C*_H__B8_	19,668.59	12,978.83		14,171.02	
σ_HB_ (e/Å)	7.01 × 10^–3^	7.03 × 10^–3^	1.15 × 10^–2^	7.70 × 10^–3^	1.01 × 10^–2^
AAD	0.2537	0.2855	0.4085	0.5124	0.7186
*R* ^2^	0.9742	0.9742	0.9422	0.9194	0.8866

a Hydrogen bond parameters (*C*_HB_) are given in kcal mol^–^^1^ Å^4^ e^–^^2^.

**Figure 2:**
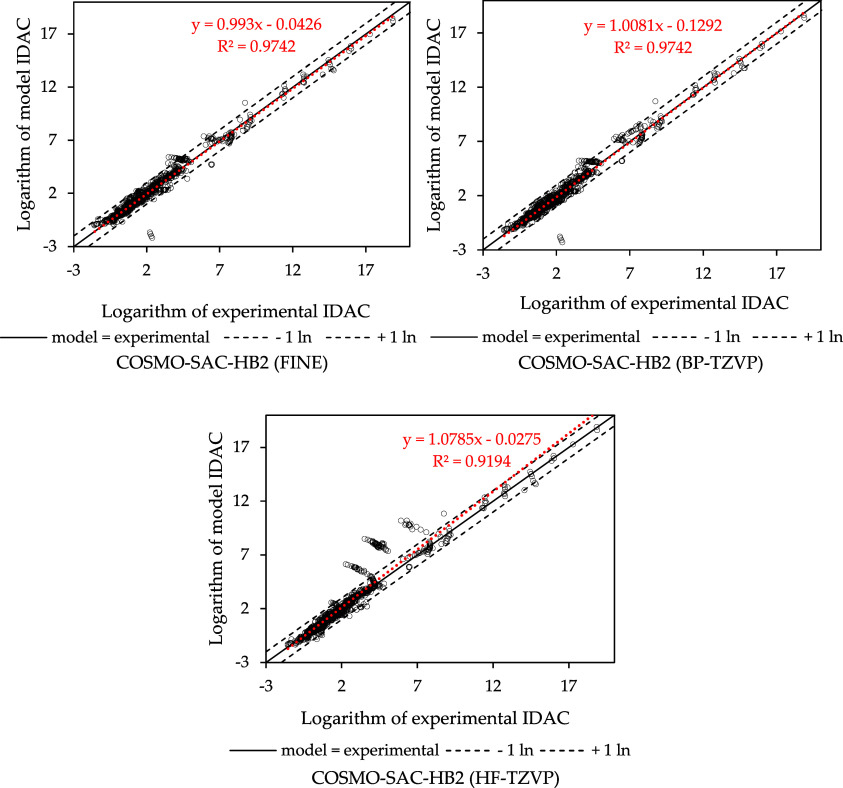
IDAC parity plots for the data used in the fitting process.

**Figure 3:**
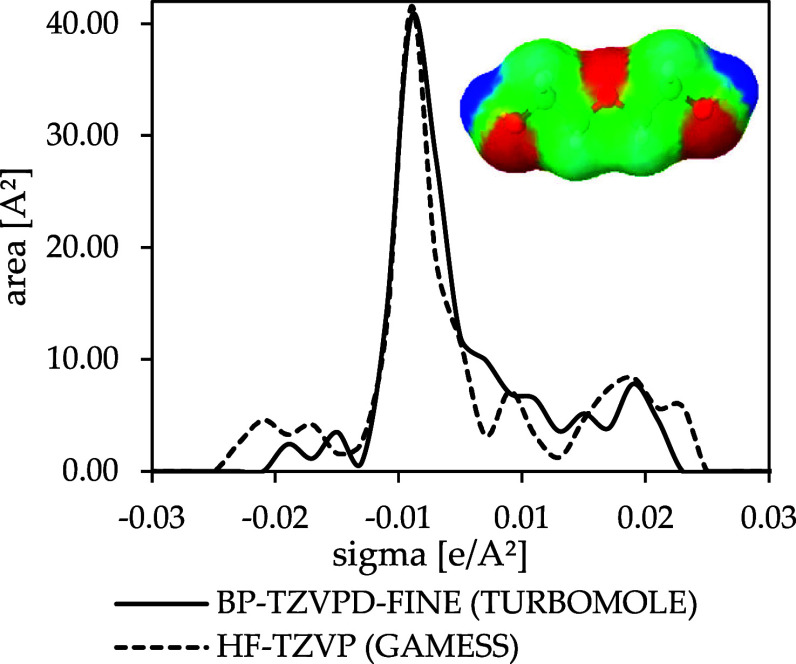
σ-Profiles for diethylene glycol obtained with FINE and HF-TZVP.

**Figure 4:**
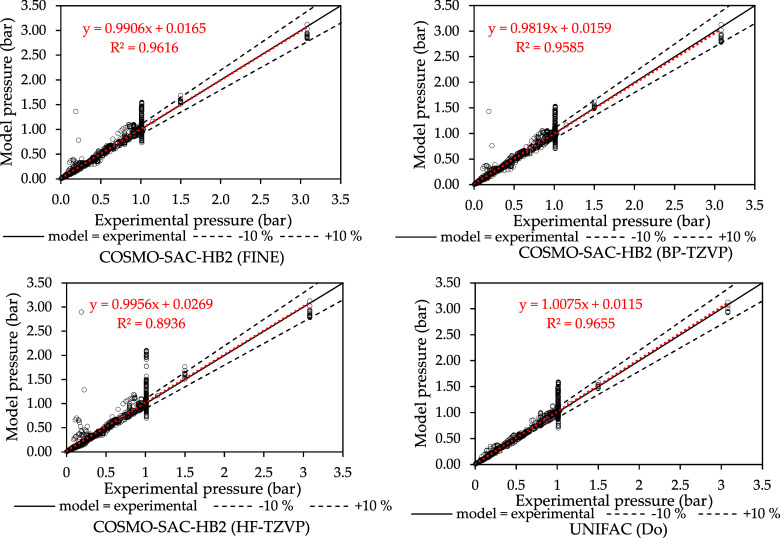
VLE pressure parity plots, experimental data taken from Danner and Gess' database.^43^

**Figure 5:**
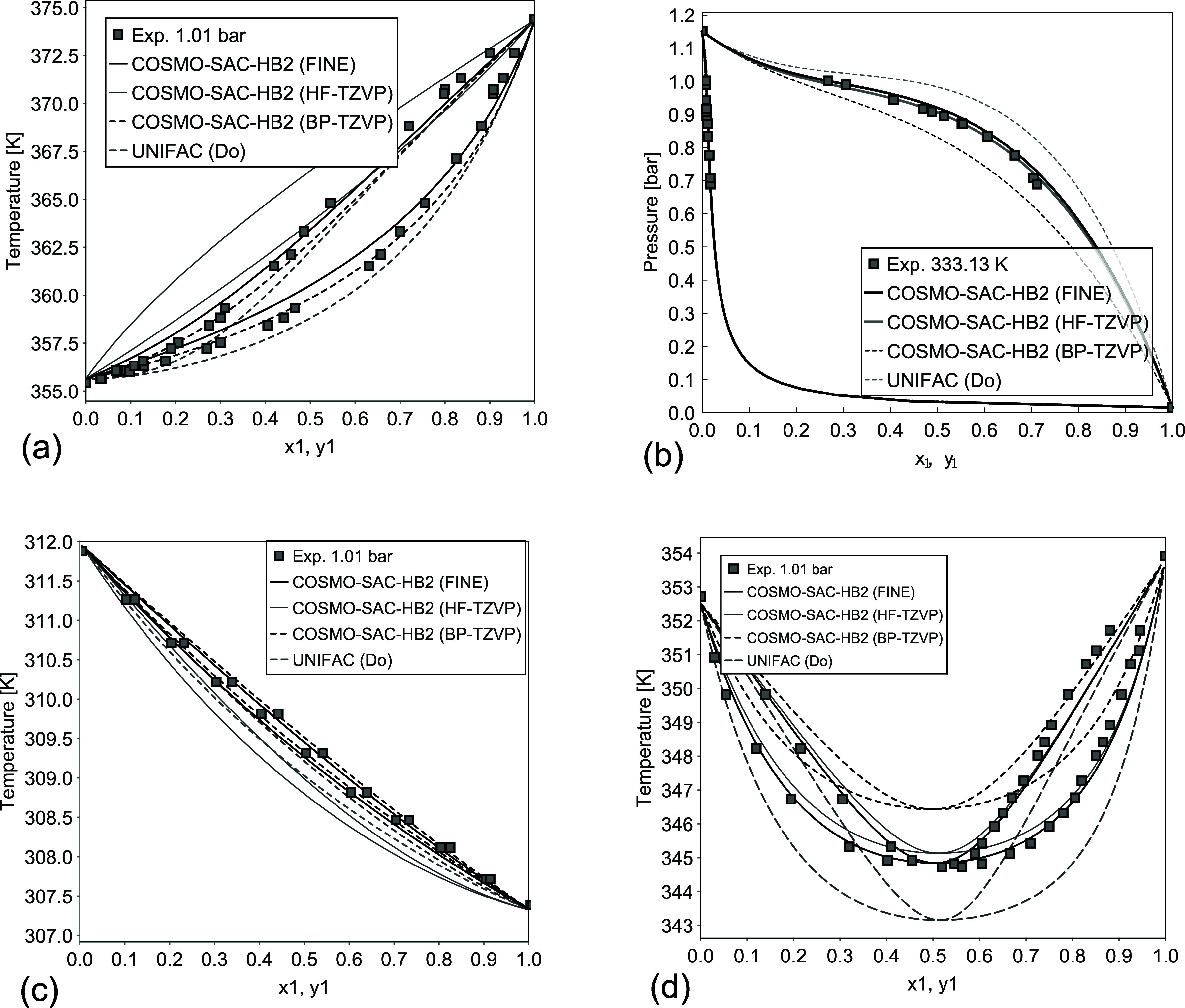
VLE prediction results using COSMO-SAC, which does not have any binary parameters, for several systems: (a) 1,4-dioxane (1)/isopropanol (2) at 101 kPa; (b) *n*-decane (1)/acetone (2) at 333 K; (c) isoprene (1)/2-methyl-2-butene (2) at 101 kPa; (d) cyclohexane (1)/methyl-ethyl-ketone (2) at 101 kPa. Experimental data taken from Danner and Gess.^43^

**Figure 6:**
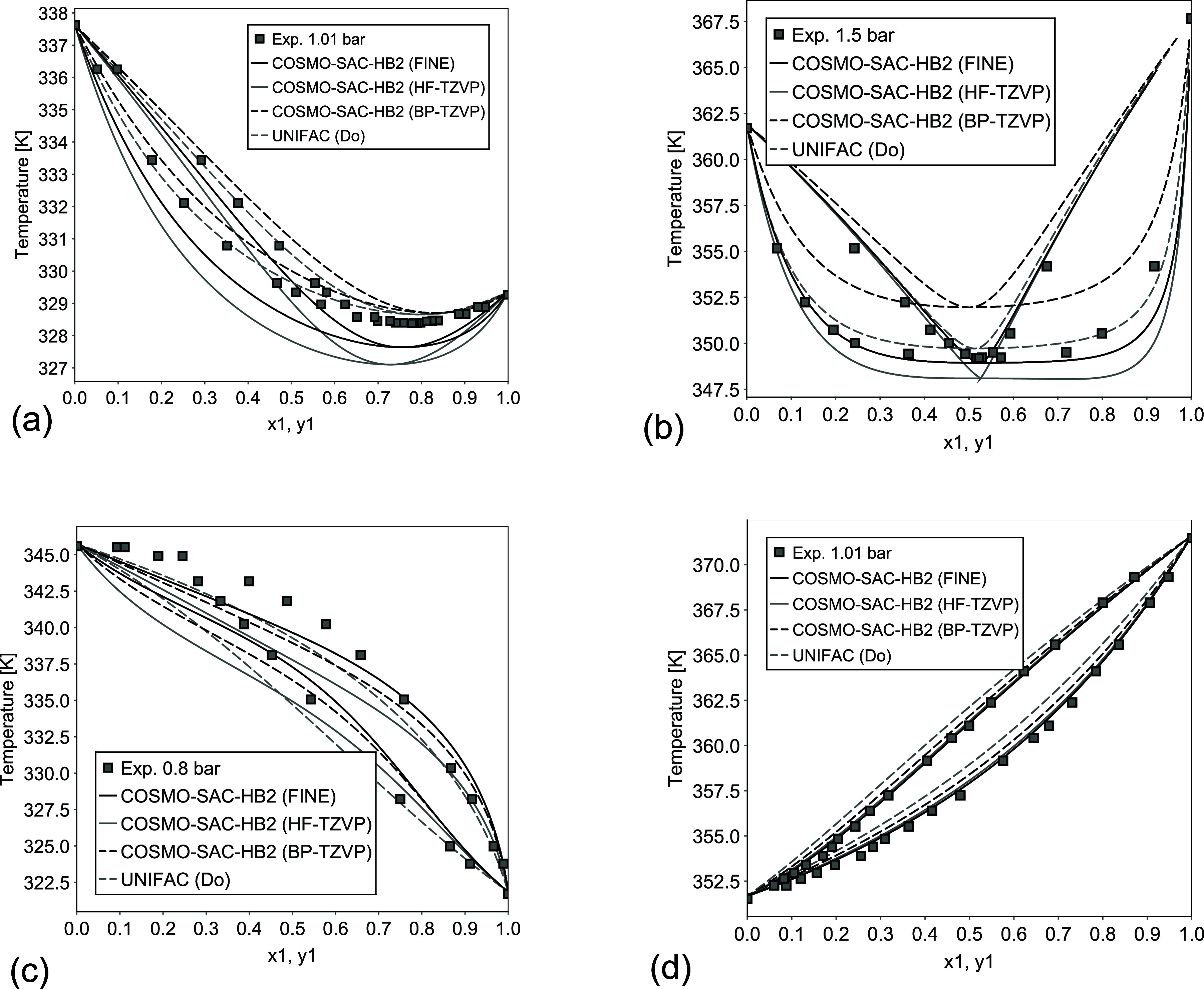
Examples of VLE prediction for different systems: (a) acetone (1)/methanol (2) at 101 kPa; (b) cyclohexane (1)/ethanol (2) at 150 kPa; (c) diethylamine (1)/ethanol (2) at 80 kPa; (d) *n*-heptane (1)/1-chlorobutane (2) at 101 kPa. Experimental data taken from Danner and Gess.^43^

**Figure 7:**
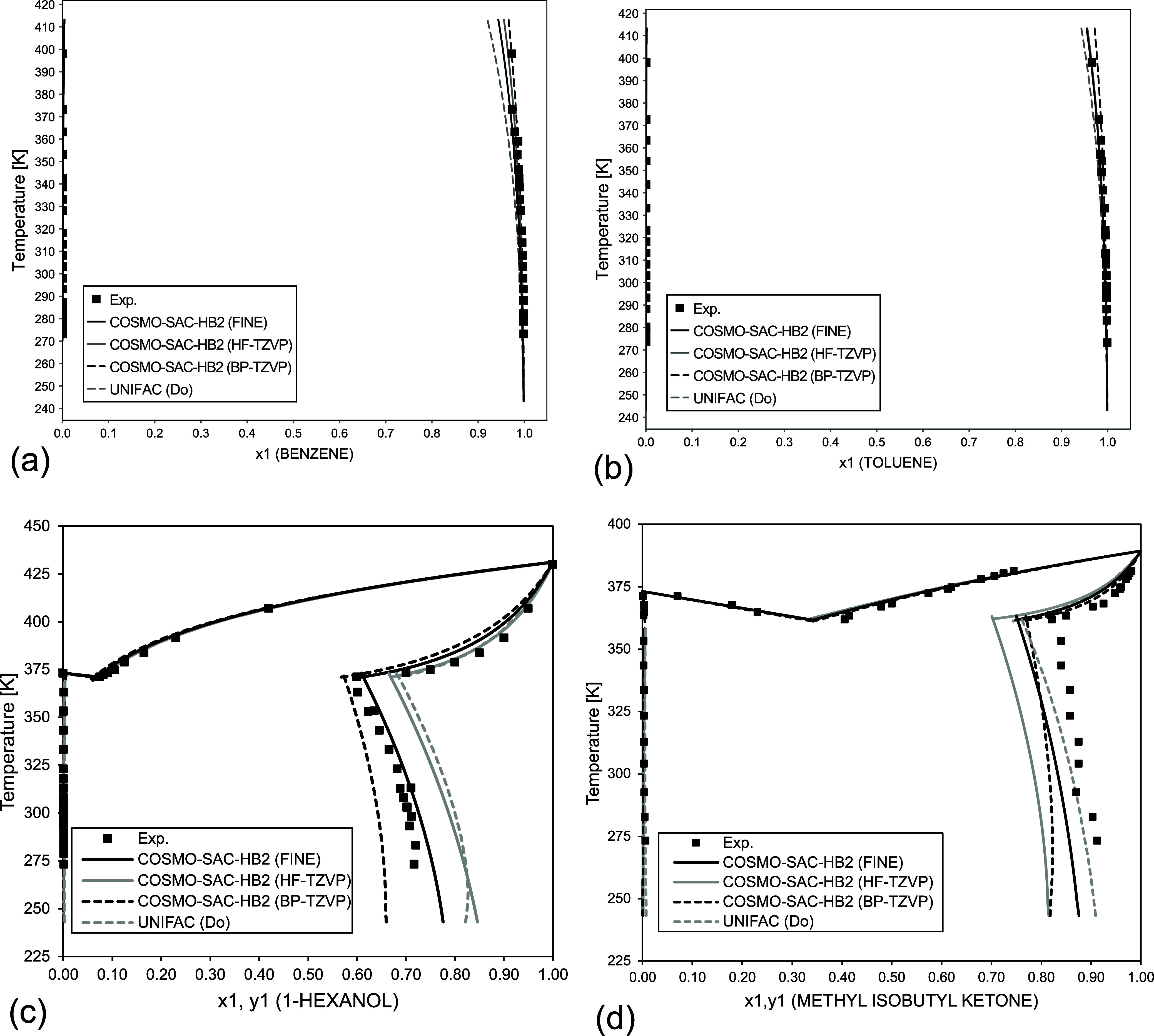
LLE diagrams for binary mixtures predicted by COSMO-SAC-HB2 and UNIFAC (Do): (a) benzene (1)/water (2) from 1 to 6 bar; (b) toluene (1)/water (2) from 1 to 4 bar; (c) 1-hexanol (1)/water (2) at 1 bar; (d) methyl isobutyl ketone (1)/water (2) at 1 bar. Experimental data taken from Goral et al. and Stephenson.^58–61^

**Figure 8:**
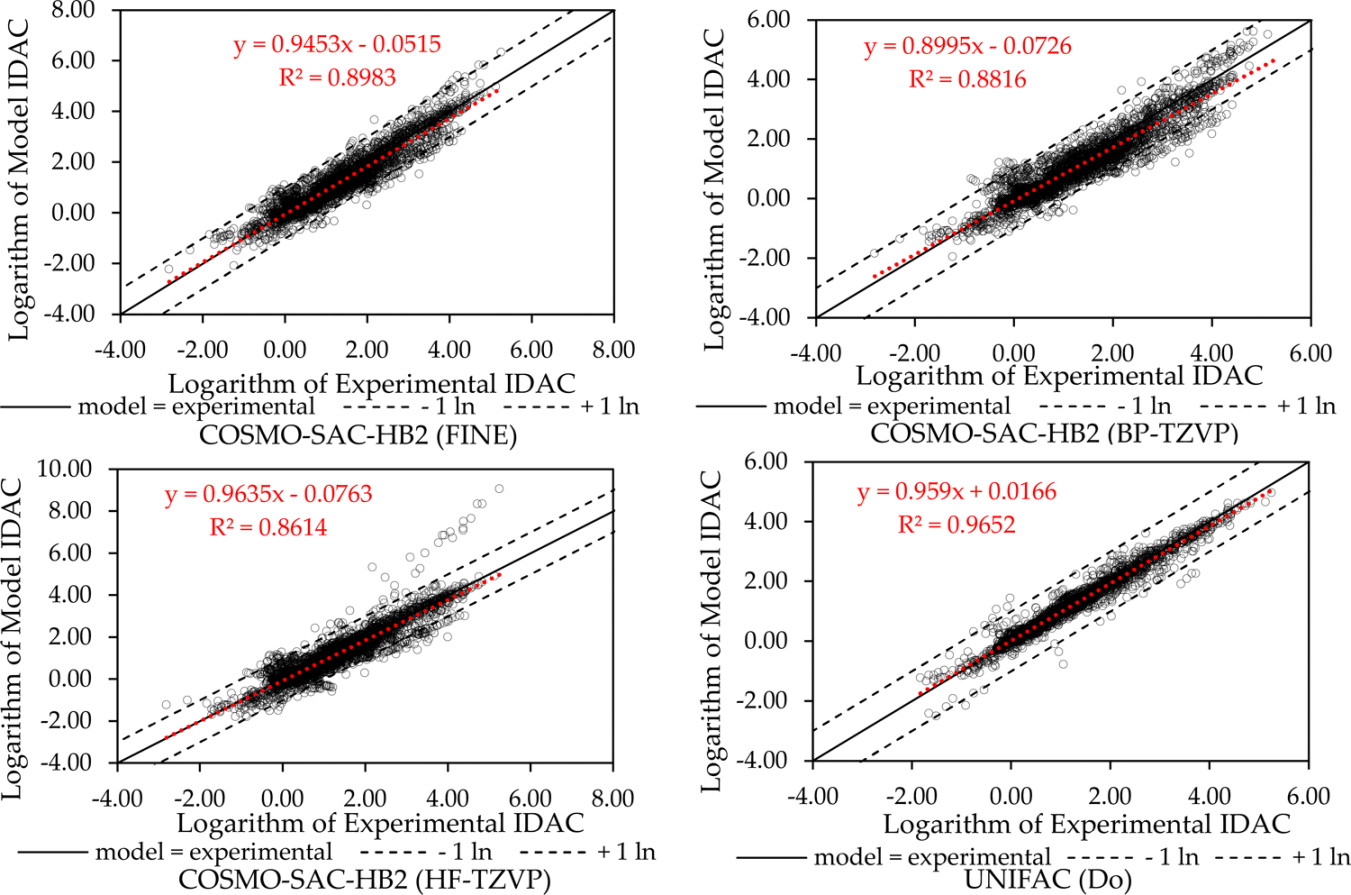
IDAC parity plots for nonaqueous systems.

**Figure 9:**
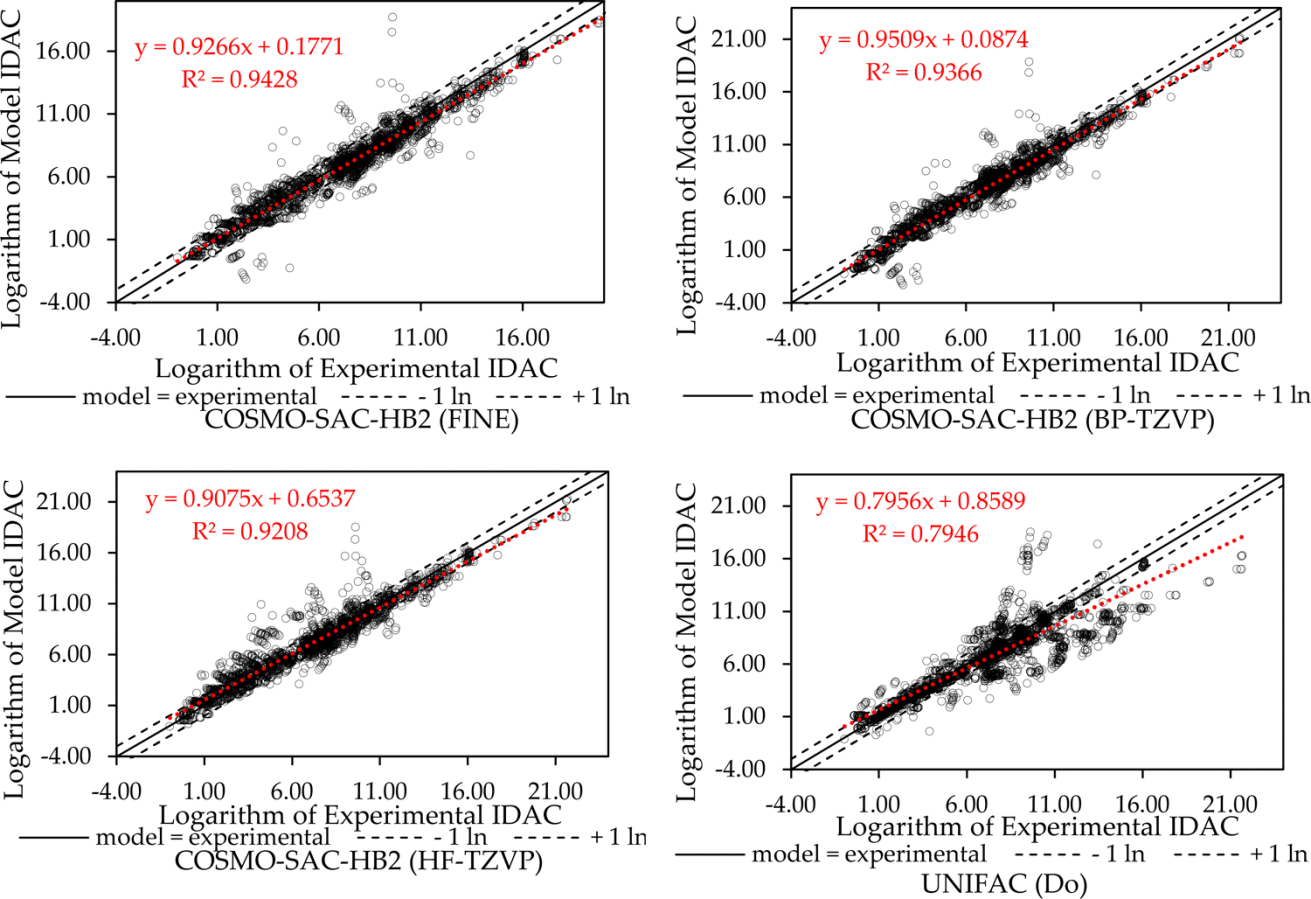
IDAC parity plots for aqueous systems.

The σ-profile of a molecule is determined by its electronic structure, which is estimated through quantum chemistry. Several computational methods can be used for this purpose, including the Hartree–Fock (HF) method and its variations that account for electronic correlation and quantum density functional theory (DFT) calculations. The goal of HF-based approaches is to approximate the solution of the Schrödinger equation for interacting all-electron systems.^17^ This yields the molecular electronic wave function (Ψ­(*r*)), which can be used to extract quantitative information, such as the σ-profile.^18^

Another alternative, implemented in TURBOMOLE software,^19^ utilizes DFT computations with the Becke^20^ and Vosko, Wilk, and Nusair (VWN) correlation of local density approximation (LDA) + Perdew^21^ functional (BP) and the triple-zeta valence polarization basis set with diffuse functions (BP-TZVPD).^22^ The TZVPD basis set is a property-optimized, diffuse-augmented extension of the Karlsruhe def2-TZVP basis set.^23^ It was constructed by variationally maximizing atomic HF polarizabilities, thereby ensuring an optimal description of molecular response properties such as linear and nonlinear polarizabilities. TZVPD uses the def2 family of basis sets as its foundation, which are known for their segmented contraction schemes, broad elemental coverage, and excellent balance between computational efficiency and accuracy in both HF and DFT calculations. By incorporating a minimal number of carefully optimized diffuse functions, the TZVPD achieves rapid and systematic convergence to the basis set limit while maintaining numerical stability. This combination makes it particularly well-suited for large-scale molecular systems where diffuse electron density and response properties are significant, without incurring the computational costs and instabilities commonly associated with heavily augmented basis sets.^24^ Along with TZVPD, TURBOMOLE also has the option to create molecular surface cavities using the fine grid marching tetrahedron cavity,^25^ which is called FINE. This method creates a COSMO surface whose segments are more uniform and evenly distributed compared to the standard COSMO cavity. This configuration corresponds to the BP-TZVPD-FINE parametrization of COSMO-RS, used in COSMOThermX software.^26^

In general, DFT computation's aim is to determine a three-dimensional function known as the electronic density distribution (*n*(*r*)), from which properties can be extracted.^27^ Neither Ψ­(*r*) nor *n*(*r*) functions have an analytical form and are typically described as linear combinations of mathematical functions, such as Gaussians and plane waves, known as basis sets.^18^ Each quantum mechanical (QM) method, along with its variants, possesses unique characteristics and employs different levels of approximation. The accuracy of the predictions made by these methods and the processing cost are highly influenced by the theory used and the quality of the basis sets used for function expansion. Consequently, the choice of the QM method and its configurations significantly impacts the performance of COSMO models, as demonstrated by Khandogin et al.^28^

In the literature, Franke and Hannebauer^29^ used various QM techniques and the COSMO-RS model to determine infinite-dilution activity coefficients (IDACs). The accuracy of the predictions was verified by comparing them to experimental data for 375 IDAC data points across multiple species. The authors concluded that the QM method affects the COSMO-RS predictions. Chen et al.^30^ also used vapor–liquid equilibrium (VLE) and liquid–liquid equilibrium (LLE), and they found out that the COSMO-SAC model is significantly sensitive to the QM method used.

In the work of Ferrarini et al.,^31^ many combinations of basis sets and levels of theory were compared, and a freely available and extensible database of σ-profiles was created. Their analysis determined that the HF-TZVP basis set was the most suitable at the time, and they developed the database using the GAMESS^32^ quantum chemistry package. Later, this database was used in JCOSMO^33^ software for the prediction of a series of equilibrium data in various applications.^11,34–36^

This study presents the first parametrization developed and implemented using the COSMO-SAC (2002)^10^ and COSMO-SAC-HB2 ^33^ models in combination with BP-TZVPD-FINE QM calculations, performed with TURBOMOLE software.^19^ The classic BP-TZVP method, widely applied with TURBOMOLE and COSMO-RS,^37–39^ is also incorporated in COSMO-SAC-HB2. The results are compared with those obtained using the database and COSMO-SAC parameters from Ferrarini et al.^31^ using the GAMESS chemistry package and the GMHB1808 ^40^ parametrization from JCOSMO using COSMO-SAC-HB2 model that applied HF-TZVP. Also, JCOSMO software is adapted to handle the TURBOMOLE files, presenting a new updated version to use .cosmo files to predict phase equilibria with any available model in JCOSMO. This paper is organized as follows. Section 2 describes the computational approach, parameter estimation, systems analyzed, and data sources used. Section 3 presents and discusses the model predictions in comparison with experimental data. Finally, Section 4 concludes the paper by summarizing the main findings and contributions.

## 2 Computational Details

The impact of the two QM approaches and basis sets on the estimation of the electronic structure was assessed. COSMO universal parameters were re-estimated for the BP-TZVPD-FINE, and the database of Ferrarini et al.^31^ was used for the HF-TZVP. Both methods tested utilize the classic triple-zeta valence polarization basis set (TZVP).^41^ The FINE approach also considers diffuse functions and the fine grid marching tetrahedron cavity.^25^

The accuracy of these methodologies was evaluated by comparing the predicted IDAC value with the experimental IDAC data collections from the Github repository of The Properties of Gases and Liquids, 6ed (PGL6ed).^42^ The full IDAC database consists of 6977 IDAC experimental data points containing a variety of substances. Additionally, VLE data from the Danner and Gess database^43^ were used to evaluate the models, and some LLE diagrams were constructed. For the assessment, the logarithm of the experimental IDAC values versus the predicted values by the model was plotted. Additionally, the UNIFAC Dortmund (Do)^7^ model was also used to estimate IDAC values for comparison. The same procedure was performed for the equilibrium pressure of the VLE data from the Danner and Gess database.^43^

In order to evaluate the performance of each model and parametrization, using the VLE database of Danner and Gess,^43^ only low to moderate pressure experiments were considered, so the modified Raoult‘s law^44^ can be used. The database is already limited to low pressures by the authors with a maximum of 3 bar. For the LLE data, temperatures corresponding to pressures of up to 6 bar were selected. Therefore, the highest temperatures in the diagram, where elevated pressures are required to maintain equilibrium, were omitted. The deviations in the equilibrium pressure (*P*) and vapor phase composition (*y*) were calculated as

$$\Delta P\%=\frac{100}{\mathrm{NP}}\sum_{i}^{\mathrm{NP}}\frac{\left|P_{i,\exp}-P_{i,\mathrm{model}}\right|}{P_{i,\exp}}$$ 1

$$\Delta y\%=\frac{100}{\mathrm{NP}}\sum_{i}^{\mathrm{NP}}|y_{i,\exp}-y_{i,\mathrm{model}}|$$ 2

where NP is the number of experimental points.

The modified Raoult‘s law and the gamma–gamma method^44^ were applied to VLE and LLE calculations, respectively. For the pure compound vapor pressures, correlations freely available in the literature were used.^45^

### 2.1 Universal Parameter Optimization

A small subset of compounds from different chemical groups was chosen for the fitting process of the COSMO-SAC universal parameters. This subset consists of hydrocarbons, esters, ethers, and amines, among others. Those compounds were chosen according to the methodology of the open and extensible σ-profile database of our group,^46^ and the list is shown in Table 1.

The objective function defined for the estimation of the COSMO-SAC universal parameters was the average absolute deviation (AAD), between the natural logarithm of calculated IDAC and the experimental data:

$$\mathrm{AAD}=\frac{1}{\mathrm{NP}}\sum_{i}^{\mathrm{NP}}|\ln\gamma_{i,\mathrm{mod}}^{\infty }-\ln\gamma_{i,\exp}^{\infty }|$$ 3

where $\ln\gamma_{i,\mathrm{mod}}^{\infty }\;\text{and}\;\ln\gamma_{i,\exp}^{\infty }$ are the IDAC predicted by the model and the experiment, respectively. The objective function was minimized using the Nelder–Mead method.^47^ The values presented by Ferrarini et al.^31^ were used as the initial estimates. The value of the average radius (*r*_avg_) was optimized for BP-TZVPD-FINE, and the optimal *r*_avg_ found by Ferrarini et al.^31^ was used for the HF-TZVP.

The global parameters of COSMO-SAC adjusted were the standard radius (*r*_eff_), the polarization factor (*f*_pol_), the cutoff value for hydrogen-bonding interactions (σ_HB_), and the constant for the hydrogen-bonding interaction (*C*_HB_). The *r*_avg_ optimization was achieved indirectly, by varying it from 0.5 to 1.8 Å while re-estimating all other parameters.

Two different versions of the COSMO-SAC model were used in this work. The first was the original COSMO-SAC model presented by Lin and Sandler,^10^ with the only modification being the inclusion of *r*_avg_ introduced by Mullins et al.^48^ The second version utilized was the COSMO-SAC-HB2,^33^ which incorporates multiple hydrogen-bonding constants.

### 2.2 Molecule Optimization and σ-Profile Generation

For the calculation of σ-profiles, two different computational chemistry packages were used. The first one is the GAMESS package, which is distributed freely. This package was utilized to perform calculations with the HF-TZVP basis set, according to previous work.^31^ The second package used is TURBOMOLE software,^19^ which is licensed by BIOVIA. This program was used to perform the calculation with the BP-TZVPD-FINE and BP-TZVP basis set. The BP-TZVPD-FINE method, a recent development that was available exclusively for the COSMO-RS model,^25^ will hereafter be referred to simply as FINE.

Initially, the structure of each compound was preoptimized using the Optimize tool of AVOGADRO software.^49^ For this purpose, the universal force field (UFF)^50^ with the steepest descent^51^ algorithm was selected. This procedure was done only for the results comprising the HF-TZVP method, according to the methodology of Ferrarini et al.^31^ For the FINE and BP-TZVP, the molecules were preoptimized using TURBOMOLE v7.4 2019 ^19^ with the geometry, frequency, noncovalent, extended tight-binding (GFN2-xTB) force field. With this initial structure, the molecules were optimized and QM calculations were conducted in each package. All calculations were performed by using a single conformer per molecule. For the HF-TZVP basis set, the molecules' geometry was optimized until the largest component of the gradient was less than 1 × 10^–4^ Ha/Bohr.^31^

## 3 Results and Discussion

### 3.1 Optimization Results

The AAD results for the optimization of *r*_avg_ in COSMO-SAC-HB2 are shown in Figure 1. The minimum AAD value was observed with *r*_avg_ = 1.5 Å. Table 2 summarizes the values of the fitted global parameters for each model along with their performance metrics, including the correlation coefficient (*R* ^2^) for the experimental versus predicted IDAC parity plots.

According to the correlation coefficient and AAD values shown in Table 2, using the σ-profiles generated with FINE, it was possible to achieve a better fitting result, with calculated IDAC more closely aligned with the experimental data. In Figure 2, it is possible to observe that many systems IDAC are better represented by using FINE since the closer the points are to the diagonal, the higher the accuracy of predicted IDAC values is. The dashed lines represent a deviation of one logarithmic unit, and most of the systems are between these lines for COSMO-SAC-HB2 using FINE, which is not observed when using HF-TZVP. In terms of the objective function, COSMO-SAC-HB2 utilizing FINE achieved a 50.48% reduction in deviation compared to the GMHB1808 parametrization used by JCOSMO, which applies COSMO-SAC-HB2 with σ-profiles generated utilizing GAMESS and HF-TZVP. It is worth noting that the *R* ^2^ for COSMO-SAC-HB2 using either BP-TZVP or FINE aligned up to the fourth decimal place, indicating comparable performance between the two methods.

The effect of different quantum mechanical methods on the σ-profile is illustrated in Figure 3, which presents the three-dimensional apparent surface charges around a diethylene glycol molecule. The image of the apparent surface charges reveals induced negative charge densities around the hydrogen atoms at the two extremities of the molecule, shown in blue. Positive charge densities around the three oxygen atoms are shown in red. Neutral regions, represented in green, are observed near the carbon atoms. This three-dimensional figure is reduced to a two-dimensional σ-profile, where the intensity varies depending on the color of the corresponding regions. Although the profiles predicted by various QM methods and basis sets appear to be qualitatively alike, there are considerable quantitative differences. However, this direct comparison is not suitable for evaluating the predictive performance. Although σ-profiles provide valuable insight into molecular surface charge distributions, they do not directly reflect the predictive power of a given QM method. Thus, the properties of several nonideal mixtures are predicted, and experimental data are used for comparison in the next sections.

### 3.2 Vapor–Liquid Data Prediction

Figure 4 shows the parity plots for the VLE pressure for all systems of the Danner and Gess^43^ database. Equilibrium calculations with the σ-profiles generated using FINE resulted in the highest correlation coefficient and a greater qualitative agreement with the experimental VLE pressure data. The parametrization done with FINE showed a greater improvement in methanol/acetone equilibria and other systems containing amines, ethers, and dipolar aprotic solvents. This is expected since Klamt and Diedenhofen^25^ specify the deficiencies of the original COSMO cavity construction for these types of compounds compared to the FINE method. Additionally, the COSMOThermX software manual, which implements FINE for COSMO-RS, emphasizes the enhanced property prediction for these types of systems. Table S1 of the Supporting Information details the deviations for equilibrium pressure and vapor composition for all systems considered. Most of the systems exhibited lower deviations for both equilibrium pressure and vapor phase composition when using FINE, with average deviations reduced by 24.78% for pressure and 4.8% for vapor composition when comparing COSMO-SAC-HB2 using HF-TZVP. The BP-TZVP produced comparable results with the FINE method but with slightly lower AAD for both Δ*y* and ΔP. This trend, indicating comparable or superior accuracy of BP-TZVP, has also been reported in the literature, with extensive studies on the most suitable applications of each method.^52,53^ Although UNIFAC (Do) yielded the best overall results, expected due to its use of binary interaction parameters, COSMO-SAC-HB2 with FINE outperformed UNIFAC (Do) for 30 out of the 103 VLE systems studied, despite not utilizing any binary parameters. The results for systems containing acids were excluded from the average results in Table S1, as the COSMO models tend to exhibit larger deviations for these systems due to dimerization effects, which would skew the comparison. Furthermore, halogens are not currently computed by our methodology using GAMESS, so σ-profiles could only be generated with TURBOMOLE for those systems.

Figure 5 showcases examples of VLE predictions using σ-profiles generated with TURBOMOLE. For comparison, results from HF-TZVP utilizing GAMESS are also included. Figure 5a illustrates a system containing a cyclic ether, which typically poses a challenge for COSMO models, especially when mixed with highly polar solvents such as water or alcohol due to hydrophobic or cooperative effects.^54^ The use of FINE improved the prediction for this type of system, although as this effect strength increases, its influence on phase equilibria grows, and the model, while improved, still exhibits high deviations, as seen in the diethylamine/water mixture. Figure 5b,d shows examples of hydrocarbons mixed with dipolar aprotic solvents, where FINE provides better agreement with experimental data. Figure 5c presents a nearly ideal system, where the COSMO-SAC-HB2 with either FINE or BP-TZVP parametrizations precisely represent the experimental data, whereas the HF-TZVP exaggerates the deviation from ideality. Additionally, the COSMO-SAC-HB2 using FINE improved the results for systems with strong hydrogen bond influence compared to the HF-TZVP, with a better description of azeotropes, as shown in Figure 6a,b. For these specific mixtures, UNIFAC (Do) had a higher deviation from experimental data but still correlated very well with most systems studied. Furthermore, Figure 6c,d presents additional VLE calculations for systems involving alcohol–amine hydrogen bonding and weakly interacting nonpolar components.

Besides these improvements in VLE prediction, our COSMO-SAC framework using TURBOMOLE remains limited when describing VLE systems where dispersion interactions dominate, such as certain refrigerant mixtures involving perfluorinated compounds. While the model can capture azeotropic behavior in mixtures with strong polar–nonpolar interactions, it fails in cases where the σ-surfaces are nearly neutral, and dispersion forces drive the nonideal behavior. This highlights the need for incorporating dispersion-sensitive corrections in future developments. Additionally, the prediction of mixing enthalpies remains a significant limitation.

### 3.3 Liquid–Liquid Data Prediction

Figure 7 presents LLE predictions using the COSMO model and UNIFAC (Do). Figure 7a,b represents systems with low miscibility (aromatics/water). In general, these systems are well-represented in the lower temperature region of the data but tend to overestimate the composition and fail to capture the effect of decreasing solubility with a temperature increase, particularly for the aromatic-rich phase. This effect is particularly known when the temperature is increased to the critical point, where it is not possible to correctly predict the data without any renormalization method,^55,56^ which is out of the scope of this work. Overall, COSMO-SAC-HB2 using FINE achieved a better result when compared with HF-TZVP, yielding predictions closer to experimental data and UNIFAC (Do) results, without any binary parameter.

Figure 7c,d shows the VLE and LLE diagrams for 1-hexanol and methyl isobutyl ketone in water. In the 1-hexanol/water system, the COSMO-SAC-HB2 (FINE) model demonstrates closest agreement with experimental data, in all temperature ranges. For the methyl isobutyl ketone/water system, UNIFAC (Do) provides the most accurate representation of the experimental data. COSMO-SAC-HB2 (FINE) also yields reasonable results, while COSMO-SAC-HB2 (HF-TZVP) exhibited significantly higher deviations for the ketone-rich phase.

### 3.4 IDAC Prediction

The full database,^42^ comprising 6977 experimental IDAC data points, was also used to evaluate the prediction for systems not used in the fit of global parameters. Figure 8 displays the IDAC parity plots for nonaqueous systems. The UNIFAC (Do) has the overall best results but struggles with systems containing multiple aromatic rings. It occurs probably because of the poor dispersion term, and the same happened with the COSMO models. The FINE method improved the result for those systems but still needs improvements to achieve UNIFAC similar results.

Figure 9 shows the IDAC parity plots for the aqueous systems. Higher deviations were noted in systems with amines and ethers in water, particularly at lower temperatures, even when using UNIFAC (Do). This is likely caused by cooperative effects and the limitations of assuming pairwise additive interactions.^54^ Among all of the COSMO-SAC versions tested, the COSMO-SAC-HB2 using FINE had the best correlation coefficient and qualitative agreement with experimental data. Most of the data exhibit deviations within the limits of one logarithmic unit (shown by dashed lines), either positive or negative. The data outside this range are from amines and ethers in water, which have very strong cooperative effects, as mentioned previously. The AAD results are shown in Table 3. COSMO-SAC-HB2 using FINE had an AAD 14.57% lower than when using HF-TZVP. UNIFAC (Do) exhibits greater deviations due to the poorer IDAC prediction of aqueous systems, as well-known in the literature.^33,57^ Thus, the COSMO-SAC performed better than the modified UNIFAC for IDAC calculation of aqueous systems but still needs improvements to achieve the same result in nonaqueous systems.

**Table 3:** Deviation Results for the Entire IDAC Database

	COSMO-SAC-HB2 (FINE)	COSMO-SAC (FINE)	COSMO-SAC-HB2 (HF-TZVP)	COSMO-SAC (HF-TZVP)	COSMO-SAC-HB2 (BP-TZVP)	UNIFAC (Do)
AAD	0.4640	0.6775	0.4785	1.7457	0.4773	0.6357
R2	0.9728	0.9599	0.9617	0.8780	0.9698	0.6208

## 4 Conclusions

New parametrizations for the COSMO-SAC of Lin and Sandler^10^ and COSMO-SAC-HB2 were developed utilizing the advanced QM method BP-TZVPD-FINE, which was only available for COSMO-RS, and the classic BP-TZVP was also used for COSMO-SAC-HB2.

The COSMO-SAC parametrizations, utilizing FINE, BP-TZVP, and HF-TZVP QM methods, were evaluated by predicting values of 6977 experimental IDAC data points, a database of VLE, and some LLE diagrams. The results showed that FINE could substantially improve the COSMO-SAC results, especially for systems containing amines, ethers, and dipolar aprotic solvents. However, limitations regarding the prediction of mixing enthalpies and dispersion-driven systems remain a challenge in our COSMO-SAC framework.

Furthermore, the use of BP-TZVP and FINE allows COSMO-SAC and COSMO-RS to use the same data bank of σ-profiles and charge density distributions. Therefore, increasing the applicability of both models toward the development of innovative solutions. Additionally, this implementation allows the coupling of two user-friendly software (TURBOMOLE and JCOSMO) to allow nonthermodynamic experts to apply and test COSMO-SAC on the prediction of properties and phase equilibrium data. This implementation is expected to ease the application of COSMO-SAC and allow for the faster development of innovative technologies.

## Supplementary Material



## Data Availability

All universal parameters were presented, and an updated version of JCOSMO with these implementations is available at https://www.ufrgs.br/lvpp/download/jcosmo/.

## Notes

(Table S1) Model deviations from experimental data and references for experimental data (PDF)

## Nomenclature

**Latin Letters**
AAD: average absolute deviation
BP: Becke and Perdew functional
COSMO: conductor-like screening model
COSMO-RS: COSMO for real solvents
COSMO-SAC: COSMO segment activity coefficient
DFT: density functional theory
FINE: fine grid marching tetrahedron cavity
GFN2-xTB: geometry, frequency, noncovalent, extended tight-binding
HF: Hartree–Fock
IDAC: infinite dilution activity coefficients
LDA: local density approximation
LLE: liquid–liquid equilibrium
MD: molecular dynamics
*n*(*r*): electronic density distribution
NP: number of points
NRTL: nonrandom two-liquid
*P*: pressure
QM: quantum mechanical
*r*: radius
TZVP: triple-zeta valence polarization
TZVPD: TZVP with diffuse functions
UFF: universal force field
UNIFAC: UNIQUAC functional-group activity coefficient
UNIFAC (Do): UNIFAC Dortmund
UNIQUAC: universal quasichemical
VLE: vapor–liquid equilibrium
VWN: Vosko, Wilk, and Nusair
*y*: vapor composition
**Greek Letters**
Ψ­(*r*): molecular electronic wave function
γ: activity coefficient
**Subscripts**
*i*: component
mod: model
exp: experimental
avg: average
pol: polarization
HB: hydrogen bond
**Superscripts**
∞: infinite dilution
