# Supplementary material for: Development of a COSMO-SAC Parametrization with Advanced QM Method TZVPD-FINE
Source: Ind Eng Chem Res. 2025 Jul 14;64(29):14700–11. doi: 10.1021/acs.iecr.5c01146 (PMC12821137; doi:10.1021/acs.iecr.5c01146)
Supplement: Supplementary file 1 [file ie5c01146_si_001.pdf]

Supporting Information

Development of a COSMO-SAC parameterization with advanced QM method TZVPD-FINE

Edgar T. de Souza Jr.[a], Murilo L. Alcantara[b], Paula B. Staudt[a], João A. P. Coutinho[b*], Rafael de P. Soares[a*]

[a]*Virtual Laboratory for Properties Prediction (LVPP), Chemical Engineering Department, Federal University of Rio Grande do Sul, Rua Ramiro Barcelos, 2777, CEP 90035-007, Porto Alegre, RS, Brazil.*

[b]*CICECO - Aveiro Institute of Materials, Chemistry Department, University of Aveiro, Aveiro, 3810-193, Portugal*

*Corresponding author at: CICECO - Aveiro Institute of Materials, Chemistry Department, University of Aveiro, Aveiro, 3810-193, Portugal
*Corresponding author at: Virtual Laboratory for Properties Prediction (LVPP), Chemical Engineering Department, Federal University of Rio Grande do Sul, Rua Ramiro Barcelos, 2777, CEP 90035-007, Porto Alegre, RS, Brazil.
Email addresses: jcoutinho@ua.pt (João A. P. Coutinho), rafael.pelegrini@ufrgs.br (Rafael de P. Soares).
Tel.: +351 234 370 200 (João A. P. Coutinho), +55 (51) 3308 2854 (Rafael de P. Soares).

The supporting information contains one table as follows:

1. Table S1 lists the ΔP % and Δy %results for all Danner and Gess [1] database of vapor-liquid equilibria. Where ΔP % and Δy % are given by:

$$\Delta P\% = \frac{100}{\mathrm{NP}} \sum_{i}^{\mathrm{N}P} \frac{|P_{i,\exp} - P_{i,\,\mathrm{model}}|}{P_{i,\exp}} \tag{1}$$

$$\Delta y\% = \frac{100}{\mathrm{NP}} \sum_{i}^{\mathrm{NP}} |y_{i,\exp} - y_{i,\,\mathrm{model}}| \tag{2}$$

Table S1 - Average percentage deviations for each vapor-liquid equilibrium system using different COSMO-SAC versions or parametrizations. Results from UNIFAC Dortmund are also included for comparison.

Model	COSMO-SAC-HB2 (FINE)		COSMO-SAC (FINE)		COSMO-SAC-HB2 (HF-TZVP)		COSMO-SAC (HF-TZVP)		COSMO-SAC-HB2 (BP-TZVP)		UNIFAC (Do)	
System	ΔP %	Δy %	ΔP %	Δy %	ΔP %	Δy %	ΔP %	Δy %	ΔP %	Δy %	ΔP %	Δy %
1,4-dioxane (1)/ isopropanol (2)	1.85	1.26	2.04	1.37	7.66	3.81	8.54	4.22	2.33	1.55	2.63	1.16
diethyl ether (1)/ dichloromethane (2)	1.38	0.74	1.28	0.71	2.48	0.95	5.51	2.04	1.70	0.75	0.84	0.69
diethyl ether (1)/ iodomethane (2)	11.14	*	10.85	*	**	**	**	**	11.03	*	0.76	0.00
ethyl acetate (1)/ isopropanol (2)	9.32	1.43	14.54	3.16	8.50	1.26	16.12	3.70	8.41	1.40	11.51	1.82
diethyl ether (1)/ chloroform (2)	4.86	2.24	5.86	1.12	1.60	0.83	14.84	3.75	2.45	1.36	2.26	1.13
n-butyl acetate (1)/ n-butanol (2)	2.00	1.18	4.48	1.38	1.70	0.97	4.95	1.51	2.57	1.51	3.10	0.64
furfural (1)/ ethanol (2)	11.46	3.67	20.04	6.47	7.33	2.68	17.16	5.28	10.04	3.19	1.93	0.92
ethyl propyl ether (1)/ chloroform (2)	2.40	0.68	5.54	1.83	1.51	0.75	11.14	3.76	0.69	0.54	0.86	0.44
n-propyl acetate (1)/ n-propanol (2)	1.70	1.43	5.49	1.55	1.10	1.05	8.04	2.48	1.81	1.49	2.17	0.12
diethyl ether (1)/ ethanol (2)	8.14	0.73	16.46	1.98	18.18	2.32	21.03	2.84	1.38	0.16	4.18	0.64
1,4-dioxane (1)/ methanol (2)	9.78	3.09	10.72	3.59	21.13	8.38	19.43	7.53	3.04	1.29	4.99	1.45
acetone (1)/ chloroform (2)	3.10	1.35	8.13	2.17	1.25	0.40	17.85	5.51	1.23	0.27	0.95	0.28
acetone (1)/ methanol (2)	2.46	1.07	10.05	4.31	4.29	1.77	3.37	1.85	7.32	2.75	0.71	0.53
water (1)/ methanol (2)	1.36	1.28	2.93	1.94	3.41	2.22	4.61	1.16	7.21	2.29	1.08	1.02
water (1)/ ethanol (2)	3.19	1.49	3.72	1.57	1.92	1.39	9.73	4.65	8.53	3.68	0.42	0.41
water (1)/ pyridine (2)	18.58	7.87	20.06	11.35	25.38	10.14	16.96	7.40	13.28	6.04	5.14	1.96
water (1)/ diethylamine (2)	84.82	10.40	38.12	17.34	159.43	11.54	24.53	7.01	87.21	10.96	7.71	1.40
water (1)/ isopropanol (2)	4.32	2.22	5.30	2.31	4.35	2.92	13.90	6.90	8.46	3.55	1.89	1.11
carbon tetrachloride (1)/ methyl ethyl ketone (2)	2.50	1.61	1.30	1.14	1.63	1.25	2.91	0.76	0.59	0.83	0.62	0.73
hexafluorobenzene (1)/ diisopropyl ether (2)	7.34	*	7.55	*	13.02	0.00	8.99	0.00	8.83	0.00	0.72	0.00
benzene (1)/ acetone (2)	4.57	1.34	4.66	1.37	6.32	2.02	6.41	2.11	7.84	2.64	3.15	0.84
toluene (1)/ methyl isobutyl ketone (2)	0.99	0.98	1.02	0.97	2.80	1.58	1.37	1.12	2.00	1.31	0.96	0.91
benzene (1)/ thiophene (2)	0.50	0.16	0.53	0.17	0.31	0.08	0.49	0.15	0.59	0.19	0.29	0.07
toluene (1)/ methyl n-propyl ketone (2)	1.70	0.79	1.71	0.78	2.64	1.76	1.19	1.09	1.81	1.40	2.25	0.69
benzene (1)/ methyl ethyl ketone (2)	4.81	0.70	4.94	0.75	5.80	1.17	5.17	0.85	5.84	1.19	4.57	0.60

Continue on the next page.

Model	COSMO-SAC-HB2 (FINE)		COSMO-SAC (FINE)		COSMO-SAC-HB2 (HF-TZVP)		COSMO-SAC (HF-TZVP)		COSMO-SAC-HB2 (BP-TZVP)		UNIFAC (Do)	
System	ΔP %	Δy %	ΔP %	Δy %	ΔP %	Δy %	ΔP %	Δy %	ΔP %	Δy %	ΔP %	Δy %
n-decane (1)/ acetone (2)	1.75	0.12	1.48	0.13	0.81	0.12	19.49	0.29	7.49	0.15	8.63	0.16
carbon tetrachloride (1)/ furfural (2)	4.53	0.96	5.93	1.04	7.25	1.40	16.76	2.24	6.51	1.10	1.23	0.70
carbon tetrachloride (1)/ acetone (2)	3.38	2.02	2.18	1.55	2.12	1.51	4.10	1.73	0.58	0.88	1.63	0.59
cyclohexane (1)/ methyl ethyl ketone (2)	0.98	0.65	1.22	0.84	1.28	0.77	7.77	4.59	2.82	1.90	4.71	2.01
n-heptane (1)/ diethyl ketone (2)	2.07	0.95	2.43	1.05	2.37	1.01	6.93	3.05	3.89	1.66	0.64	0.49
n-heptane (1)/ thiophene (2)	3.87	1.79	4.27	1.96	1.31	0.85	6.94	3.11	3.64	1.70	5.98	2.50
n-heptane (1)/ methyl ethyl ketone (2)	2.24	0.69	3.12	0.80	2.64	0.63	10.27	3.77	5.70	1.78	3.62	2.01
n-propionaldehyde (1)/ methyl ethyl ketone (2)	1.45	0.63	1.56	0.65	1.56	0.65	1.57	0.65	1.44	0.63	2.62	0.88
acetone (1)/ vinyl acetate (2)	3.32	0.92	3.03	0.86	3.84	1.08	3.07	0.88	3.97	1.11	3.43	0.92
acetone (1)/ n-propyl acetate (2)	3.66	1.21	3.48	1.15	4.60	1.57	5.11	1.76	4.52	1.54	2.31	0.58
n-propionaldehyde (1)/ acetone (2)	1.86	0.54	1.79	0.53	1.62	0.50	1.60	0.50	1.70	0.52	2.58	0.61
acetaldehyde (1)/ methyl acetate (2)	1.99	1.15	1.87	1.22	2.27	1.11	2.41	1.09	2.76	1.06	3.37	1.54
acetaldehyde (1)/ vinyl acetate (2)	13.36	5.17	12.52	4.87	15.77	6.12	14.10	5.50	15.39	5.97	13.25	5.17
acetaldehyde (1)/ diethyl ether (2)	2.87	1.19	3.21	1.32	1.82	0.85	7.17	2.88	6.79	2.73	1.72	0.81
diethyl ether (1)/ acetone (2)	2.22	0.71	2.40	0.79	2.81	0.95	5.73	2.32	4.35	1.68	2.90	1.48
ethylbenzene (1)/ n-heptane (2)	0.20	0.51	0.14	0.50	1.68	0.81	0.77	0.38	1.08	0.69	1.03	0.67
n-heptane (1)/ p-xylene (2)	0.47	0.62	0.44	0.62	0.96	0.83	1.42	0.72	0.70	0.79	2.61	1.25
benzene (1)/ toluene (2)	1.51	0.58	1.56	0.57	1.28	0.59	1.27	0.60	1.47	0.58	1.35	0.59
carbon tetrachloride (1)/ benzene (2)	1.24	0.60	1.03	0.52	2.03	0.90	0.50	0.17	2.29	0.99	0.55	0.18
carbon disulfide (1)/ cyclohexane (2)	7.45	2.35	6.89	2.16	7.11	2.24	7.11	2.23	7.11	2.23	0.59	0.22
hexafluorobenzene (1)/ toluene (2)	2.55	0.82	2.44	0.78	1.04	0.55	1.09	0.55	2.42	0.78	1.12	0.48
isoprene (1)/ 2-methyl-2-butene (2)	0.21	0.12	0.21	0.12	1.11	0.45	0.19	0.11	0.45	0.21	0.63	0.27
1-heptene (1)/ toluene (2)	2.56	*	2.59	*	1.66	0.00	2.60	0.00	2.04	0.00	1.52	0.00
cyclohexane (1)/ toluene (2)	1.74	0.90	1.62	0.91	3.32	1.12	1.52	1.04	2.70	0.96	2.54	0.88
n-octane (1)/ ethylbenzene (2)	0.67	0.50	0.78	0.53	0.70	0.28	1.42	0.75	0.24	0.31	0.66	0.47
benzene (1)/ cyclohexane (2)	0.18	0.73	0.19	0.72	0.85	1.02	2.70	0.63	1.23	1.17	0.18	0.74
carbon disulfide (1)/ carbon tetrachloride (2)	6.79	1.88	6.34	1.74	7.00	1.95	7.19	2.02	6.73	1.86	0.68	0.48
carbon disulfide (1)/ cyclopentane (2)	6.95	2.61	6.70	2.50	6.76	2.53	6.74	2.52	6.80	2.55	0. 70	0.22

Continue on the next page.

Model	COSMO-SAC-HB2 (FINE)		COSMO-SAC (FINE)		COSMO-SAC-HB2 (HF-TZVP)		COSMO-SAC (HF-TZVP)		COSMO-SAC-HB2 (BP-TZVP)		UNIFAC (Do)	
System	ΔP %	Δy %	ΔP %	Δy %	ΔP %	Δy %	ΔP %	Δy %	ΔP %	Δy %	ΔP %	Δy %
methylcyclopentane (1)/ benzene (2)	0.20	0.27	0.17	0.27	1.29	0.53	2.42	0.97	1.35	0.54	0.77	0.43
hexafluorobenzene (1)/ cyclohexane (2)	19.40	5.64	19.34	5.62	15.21	4.15	17.62	5.00	19.02	5.50	0.21	0.95
hexafluorobenzene (1)/ p-xylene (2)	14.96	*	14.74	*	11.87	0.00	12.96	0.00	14.67	0.00	1.15	0.00
cyclopentane (1)/ chloroform (2)	1.68	0.61	2.03	0.75	0.65	0.30	3.45	1.28	1.13	0.45	0.17	0.20
toluene (1)/ nitrobenzene (2)	2.10	0.30	2.14	0.32	3.30	0.40	3.70	0.47	3.27	0.43	4.36	0.58
benzene (1)/ 1,2-dichloroethane (2)	5.72	1.59	5.72	1.59	3.09	0.61	1.83	0.38	4.20	1.02	1.62	0.42
2,3-dimethylbutane (1)/ chloroform (2)	3.52	1.44	3.51	1.44	3.06	1.26	5.15	2.10	2.92	1.21	0.96	0.43
toluene (1)/ 1,2-dichloroethane (2)	15.83	2.94	15.94	2.98	9.57	1.35	7.05	0.80	12.75	2.15	6.95	0.82
n-heptane (1)/ triethylamine (2)	1.04	0.45	0.93	0.41	0.97	0.43	0.60	0.27	0.68	0.32	0.68	0.34
benzene (1)/ diethylamine (2)	4.20	0.97	3.92	0.87	4.48	1.08	3.93	0.87	3.89	0.85	1.81	0.26
ethylbenzene (1)/ acrylonitrile (2)	7.94	1.04	8.89	1.28	4.53	0.69	4.03	1.74	4.96	0.53	11.11	1.72
n-heptane (1)/ 1-chlorobutane (2)	0.70	0.89	0.92	0.91	0.98	0.91	3.07	1.50	1.31	0.94	2.67	1.36
benzene (1)/ triethylamine (2)	3.37	0.67	3.05	0.58	4.34	1.01	4.17	0.92	2.96	0.62	0.92	0.81
benzene (1)/ tert-butanol (2)	4.66	1.30	9.92	3.39	3.60	0.97	4.63	1.35	6.07	1.63	1.62	2.14
cyclohexane (1)/ pyridine (2)	5.34	1.41	5.69	1.54	2.25	0.75	14.01	4.13	3.65	0.99	45.53	15.80
cyclohexane (1)/ ethanol (2)	1.71	1.98	6.35	1.31	4.11	3.13	5.79	1.45	2.54	1.03	1.62	1.27
benzene (1)/ isopropanol (2)	5.28	2.84	12.79	5.84	3.55	2.01	5.51	3.07	5.89	3.08	1.01	0.61
hexafluorobenzene (1)/ methanol (2)	9.58	4.31	4.87	1.47	8.66	1.17	8.91	1.57	4.42	2.63	3.95	0.89
n-heptane (1)/ iodoethane (2)	4.95	2.59	4.67	2.49	**	**	**	**	2.41	1.66	2.03	0.58
benzene (1)/ nitromethane (2)	3.74	2.18	3.76	2.18	23.06	9.45	1.26	0.53	1.05	0.76	0.60	0.36
tetrachloroethylene (1)/ ethanol (2)	5.14	2.05	10.35	3.25	4.52	1.71	6.21	1.93	5.33	2.10	3.63	1.05
n-pentane (1)/ n-butanol (2)	0.84	*	12.28	*	5.36	0.00	5.61	0.00	3.24	0.00	1.94	0.00
n-octane (1)/ methanol (2)	6.23	1.31	6.87	0.95	10.46	1.94	6.45	0.84	5.87	1.25	0.99	0.25
ethylbenzene (1)/ nitrobenzene (2)	5.42	*	5.33	*	7.61	0.00	3.40	0.00	4.11	0.00	4.65	0.00
hexafluorobenzene (1)/ n-propanol (2)	9.74	1.41	20.40	3.57	18.24	3.28	17.40	3.21	13.77	2.24	6.08	0.58
n-octane (1)/ pyridine (2)	5.77	1.96	6.01	2.01	2.42	0.93	13.60	4.90	4.48	1.60	0.45	0.97
benzene (1)/ ethanol (2)	6.30	4.39	13.50	8.25	4.18	3.37	4.61	3.61	7.95	5.26	2.15	2.13

Continue on the next page.

Model	COSMO-SAC-HB2 (FINE)		COSMO-SAC (FINE)		COSMO-SAC-HB2 (HF-TZVP)		COSMO-SAC (HF-TZVP)		COSMO-SAC-HB2 (BP-TZVP)		UNIFAC (Do)	
System	ΔP %	Δy %	ΔP %	Δy %	ΔP %	Δy %	ΔP %	Δy %	ΔP %	Δy %	ΔP %	Δy %
methanol (1)/ cyclohexane (2)	5.94	2.95	5.05	1.18	8.92	4.03	5.82	1.82	5.57	2.70	1.78	0.71
2-methylpentane (1)/ nitroethane (2)	26.36	3.07	20.25	2.54	61.66	5.29	13.74	2.40	13.58	1.86	0.41	0.24
water (1)/ cyclohexanone (2)	13.61	4.03	28.48	11.85	13.65	4.11	36.91	20.25	12.91	3.54	41.84	4.50
water (1)/ phenol (2)	12.86	1.44	32.65	8.29	8.36	0.42	29.75	3.55	18.37	2.33	5.18	0.34
n-hexane (1)/ nitroethane (2)	30.67	4.27	23.08	3.46	75.74	7.67	13.40	3.36	16.31	2.57	2.66	0.33
methanol (1)/ isobutanol (2)	5.41	0.66	4.40	0.49	5.55	0.68	7.28	1.06	5.04	0.59	4.60	0.52
ethanol (1)/ isopropanol (2)	0.33	0.26	0.31	0.25	0.26	0.24	0.34	0.27	0.24	0.24	0.82	0.42
tert-butanol (1)/ n-butanol (2)	2.35	2.56	1.85	2.38	2.41	2.58	2.39	2.58	2.38	2.57	6.76	3.86
n-propanol (1)/ isobutanol (2)	1.29	0.51	1.20	0.51	1.29	0.51	1.53	0.50	1.42	0.50	0.90	0.59
ethanol (1)/ isobutanol (2)	0.41	0.28	1.04	0.43	0.42	0.28	0.51	0.24	0.40	0.23	1.33	0.50
ethanol (1)/ triethylamine (2)	14.64	5.60	5.32	1.84	3.70	3.10	7.63	6.42	6.05	2.78	1.70	1.33
n-butylamine (1)/ n-propanol (2)	6.54	2.40	22.62	7.45	5.39	2.99	19.43	7.08	9.79	3.49	1.53	1.07
1,2-dichloroethane (1)/ isobutanol (2)	1.95	0.61	10.14	2.98	3.15	0.88	3.64	1.05	3.13	0.87	3.61	0.58
bromobenzene (1)/ cyclohexanol (2)	1.72	0.87	7.14	2.63	**	**	**	**	1.91	0.82	3.34	1.63
methanol (1)/ 1,2-dichloroethane (2)	9.44	4.99	17.29	8.57	9.26	4.81	3.56	2.78	8.95	4.95	1.82	1.87
diethylamine (1)/ ethanol (2)	5.73	2.62	17.19	7.80	8.17	3.17	9.81	4.23	6.92	2.76	5.33	1.49
ethanol (1)/ acetonitrile (2)	2.77	2.05	5.45	2.69	2.23	2.06	5.39	3.20	2.15	2.07	1.58	0.71
n-butylamine (1)/ n-butanol (2)	6.72	1.93	23.57	9.32	2.03	1.30	18.23	6.71	10.18	3.27	1.22	1.27
chlorobenzene (1)/ propionic acid (2)	19.75	11.63	11.58	8.79	29.41	14.47	32.04	14.95	16.22	10.45	4.45	6.34
n-butyl formate (1)/ formic acid (2)	14.82	4.57	2.18	2.06	52.77	16.21	9.84	2.98	13.67	4.21	15.16	3.95
formic acid (1)/ acetic acid (2)	3.36	3.75	9.04	3.59	7.94	6.06	7.29	5.92	3.19	3.78	1.88	4.15
dichloromethane (1)/ acetic acid (2)	16.10	8.69	11.47	7.73	21.01	9.32	27.27	9.87	13.12	8.14	8.61	7.62
carbon tetrachloride (1)/ acetic acid (2)	90.28	12.37	54.10	10.22	195.56	15.69	99.57	12.53	84.73	12.07	5.53	5.44
Average	6.06	1.80	7.31	2.47	8.05	1.89	7.77	2.47	5.93	1.71	3.44	1.06

*Only P-x data available.

** Limitation in our current methodology using GAMESS.

References

[1] R.P. Danner, M.A. Gess, A database standard for the evaluation of vapor-liquid-equilibrium models, Fluid Phase Equilib 56 (1990) 285–301. https://doi.org/10.1016/0378-3812(90)85109-N.
